# Reinforcement of Natural Rubber Latex Using Jute Carboxycellulose Nanofibers Extracted Using Nitro-Oxidation Method

**DOI:** 10.3390/nano10040706

**Published:** 2020-04-08

**Authors:** Sunil K. Sharma, Priyanka R. Sharma, Simon Lin, Hui Chen, Ken Johnson, Ruifu Wang, William Borges, Chengbo Zhan, Benjamin S. Hsiao

**Affiliations:** 1 Department of Chemistry, Stony Brook University, Stony Brook, NY 11794-3400, USA; sunil.k.sharma@stonybrook.edu (S.K.S.); simon.lin@stonybrook.edu (S.L.); hui.chen.2@stonybrook.edu (H.C.); ken.johnson@stonybrook.edu (K.J.); Ruifu.Wang@stonybrook.edu (R.W.); chengbo.zhan@stonybrook.edu (C.Z.); 2 Roslyn High School, Roslyn, NY 11576, USA; WBorges20@roslynschools.org

**Keywords:** natural rubber latex, NOCNF, jute fibers, nitro-oxidation

## Abstract

Synthetic rubber produced from nonrenewable fossil fuel requires high energy costs and is dependent on the presumed unstable petroleum price. Natural rubber latex (NRL) is one of the major alternative sustainable rubber sources since it is derived from the plant ‘*Hevea brasiliensis*’. Our study focuses on integrating sustainably processed carboxycellulose nanofibers from untreated jute biomass into NRL to enhance the mechanical strength of the material for various applications. The carboxycellulose nanofibers (NOCNF) having carboxyl content of 0.94 mmol/g was prepared and integrated into its nonionic form (–COONa) for its higher dispersion in water to increase the interfacial interaction between NRL and NOCNF. Transmission electron microscopy (TEM) and atomic force microscopy (AFM) analyses of NOCNF showed the average dimensions of nanofibers were length (L) = 524 ± 203 nm, diameter (D) 7 ± 2 nm and thickness 2.9 nm. Furthermore, fourier transform infra-red spectrometry (FTIR) analysis of NOCNF depicted the presence of carboxyl group. However, the dynamic light scattering (DLS) measurement of NRL demonstrated an effective diameter in the range of 643 nm with polydispersity of 0.005. Tensile mechanical strengths were tested to observe the enhancement effects at various concentrations of NOCNF in the NRL. Mechanical properties of NRL/NOCNF films were determined by tensile testing, where the results showed an increasing trend of enhancement. With the increasing NOCNF concentration, the film modulus was found to increase quite substantially, but the elongation-to-break ratio decreased drastically. The presence of NOCNF changed the NRL film from elastic to brittle. However, at the NOCNF overlap concentration (0.2 wt. %), the film modulus seemed to be the highest.

## 1. Introduction

Synthetic and natural rubber are a staple commodity for numerous industrial applications [1,2,3,4,5,6]. The International Rubber Study Group (IRSG) [7] reported that the U.S. consumed 2.7 million metric tons of rubber ranging from automotive parts to sealants in 2013. These products consist of mostly synthetic rubber derived from petroleum sources and natural rubber derived from Hevea trees (*Hevea brasiliensis*). The use of petroleum-derived synthetic rubber causes several concerns [8,9]. The reliance on nonrenewable resources causes a dependent and unstable price for synthetic rubber costs. Synthetic rubber production also requires a higher energy consumption and is environmentally intensive when compared to using natural rubber [8]. Natural rubber in its unprocessed or raw form has low strength and that limit their applications. The strength of the natural rubber latex is improved mostly by the vulcanization process, where the long chains of rubber molecule are cross-linked through the chemical process which ultimately transform the natural rubber latex into a strong elastic product (natural rubber) with reversible deformability, good mechanical strength, excellent dynamic properties and fatigue resistance [10]. The mechanical properties of natural rubber latex can be improved by addition of varying types of reinforcing fillers.

Nanocellulose is a most abundant, inexpensive and renewable nanomaterial that has potential in many different applications including pharmaceuticals, food, energy storage, water purification, biomedical, 3D printing, anti-bacterial, carbon nanotubes stabilizer, electronics and tissue engineering. Owing to its exceptional mechanical properties, nontoxicity, biodegradability and tunable chemistry of surface hydroxyl groups, nanocellulose has garnered tremendous levels of attention over the past decades [11,12,13]. There are many methods reported for preparation of nanocellulose from various biomass sources, for example, carboxymethylation, acid hydrolysis to produce cellulose nanocrystals, 2,2,6,6-Tetramethylpiperidin-1-yl)oxyl (TEMPO) oxidation, nitro-oxidation etc. 

A potential reinforcing agent for the latex rubbers is the derivatives of natural cellulose polymers [14,15,16,17,18,19]. These polymers are called carboxycellulose and have been widely used for biomedical applications such as surgical sutures [20,21,22]. Recent developments of carboxycellulose in the nanoscale have further expanded their uses in making strengthened nanocomposite materials [23,24,25]. Since carboxycellulose nanofibers is derived from cellulose microfibril building blocks, it is readily able to be extracted from a variety of biomass materials [26,27]. Some of these biomass materials include jute, spinifex, agave and agricultural wastes [28]. Our study primarily focuses on jute-derived carboxycellulose nanofibers extracted using the recently developed nitro-oxidation method [28,29,30,31,32]. The nitro-oxidation method is found to be a simple, cost-effective process to extract the carboxycellulose nanofibers from any type of raw biomass that does not require any pretreatment steps. However, the other methods—TEMPO oxidation and carboxymethylation processes—are fully efficient in extracting the carboxycellulose nanofibers from delignified pulp, which requires prior treatment of raw biomass. Nitro-oxidation produces carboxycellulose nanofibers with residual lignin and hemicellulose impurities; however, it requires less chemicals, processing time and steps for their extraction. Additionally, the unused effluent of the reaction has potential to be converted into nitrogen-rich plant fertilizer. The nitro-oxidation method involves the reaction of nitric acid with sodium nitrite to create nitroxonium ions (NO^+^) which attacks the hydroxyl group on cellulose to produce a nitrite ester (R–CH_2_–O–NO). The nitrite ester then decomposes and generates nitroxyl (HNO) and aldehyde groups which is further oxidized into carboxyl (COOH) groups. This oxidation cycle continues at the presence of excess HNO and HNO_2_ to create the saturated carboxyl groups which provide the function sites for further chemical reaction. Since this is a recently developed method [28,31,33,34,35], there are also interests in applying these carboxycellulose nanofibers materials for further testing. 

The primary focus of this study is to integrate this low-cost nitro-oxidized carboxycellulose nanofibers (NOCNF) into natural rubber latex sources to observe the enhancement effect of the samples at various concentrations. Since latex are primarily composed of cis 1,4 polyisoprene emulsions in water [36], we chose the carboxylate functional group (–COO^−^) to induce a high dispersity during the integration stage. The carboxylate functional group is hydrophilic which along with dispersity, could allow for better interfacial interactions between the fibers and the isoprene molecules. Other latex enhancement studies also indicate the use of a hydrophilic carboxycellulose nanofibers state to be effective for making enhanced nanocomposites with enhanced tensile modulus [23]. 

## 2. Methodologies

### 2.1. Materials

Untreated jute fibers were provided by Toptrans Bangladesh Ltd. (Dhaka, Bangladesh). Fibers were cut to 3–5 cm in length and further grinded by an IKA MF 10 basic grinder at 1000 rpm (IKA Works Inc., Wilmington, NC, USA). Analytical-grade nitric acid (ACS reagent 60%) and sodium nitrite (ACS reagent ≥ 97%) were purchased from Sigma-Aldrich (Allentown, PA, USA); sodium bicarbonate was purchased from Fischer Scientific (Fairlawn, NJ, United States). Processed polygen liquid latex (NRL) nonvulcanized with 60% concentration was obtained from UK suppliers (ReAgent, Runcorn, UK). 

### 2.2. Experimental Method

#### 2.2.1. Preparation of Carboxycellulose Nanofibers (NOCNF)

Fifteen grams of untreated grinded jute fibers were placed in a 3 L three-neck, round-bottom flask with 210 mL of 60 % nitric acid. Fibers in the flask were allowed to completely soak before adding 14.4 g of sodium nitrite. The addition of sodium nitrite forms red gas inside the flask due to generation of NO_2_ gas. Hence, the mouths of the round bottom flask were sealed with stoppers sealed with parafilm. The reaction was performed at 40 °C for 16 h and was then quenched by adding 1 L distilled (DI) water. The supernatant liquid was discarded to remove excess acid and decantation with 70% ethanol and DI water in the ratio of 80:20 was performed 4–5 times until the suspended fibers stopped settling down. The fibers suspension was then transferred to a dialysis bag (Spectral/Por, molecular weight cut-off (MWCO): 6–8 kDA) and equilibrated until the conductivity of the water reached below 5 μS. The fibers were then treated with 8% sodium bicarbonate up to pH 7.5, to transform the initially generated carboxyl group (COOH) to carboxylate groups (COO^−^). To remove the excess bicarbonate from the fibers, the suspension was again dialyzed using the dialysis bag until the conductivity of the water reached below 5 μS. The 0.2 wt. % of fibers suspension then passed through homogenizer (GEA Niro Soavi Panda Plus Bench top homogenizer, Columbia, MD, USA), at 250 bar for one cycle.

#### 2.2.2. Nanocomposite Preparation

A 50 mL closed glass vial was used to integrate NRL and NOCNF. Ten milliliters of 60% solid NRL was measured for each 0–0.4 wt. % sample. A 0.26 wt. % NOCNF suspension with different volume was added into NRL solution to prepare the solution containing different (0.1, 0.2 and 0.4 wt. %) of NOCNF. The solutions were set to stir for 16 h followed by 1 h of sonication. Afterwards the prepared solution was casted evenly on a petri dish and degassed to prevent bubbles forming.

#### 2.2.3. Characterization of Carboxycellulose Nanofibers (NOCNF)

##### Fourier Transform Infra-Red Spectrometry (FTIR)

The FTIR curve was measured with a PerkinElmer Spectrum One instrument (product model, city, country) with the transmission mode set between 450 and 4000 cm^−1^. Three scans were taken per sample with a resolution of 4 cm^−1^.

##### Conductometric Titration Method

The carboxylate (–COO^−^) group in NOCNF was determined by measuring the conductivity throughout a base titration experiment. A calculated volume containing 0.3 g of nanofibers was dispersed in 55 mL of DI water. The pH was set between 2.5 and 3.0 by adding 0.1 M HCl. The solution was then titrated with 0.4 M NaOH at a rate of 0.1 mL/min until pH reached 11. Throughout the titration, the pH was measured along with the conductivity. The carboxylate content was calculated using the conductivity and pH curves.

##### Lignin and Hemicellulose Analysis in Raw Jute Fibers and NOCNF

Lignin and hemicellulose (total sugar) analysis of the samples was performed by Celignis (Limerick, Ireland). The following analytical procedures were used: (1) acid hydrolysis of samples, (2) determination of acid-soluble lignin (ASL) using Ultraviolet–Visible (UV-Vis) spectroscopy, (3) gravimetric determination of klason lignin (KL) and (4) chromatographic analysis of hydrolysate. A detailed explanation of the analytical procedure is provided in Supplementary Materials.

##### Transmission Electron Microscopy (TEM)

The TEM image was obtained with a FEI Tecnai G2 Spirit Bio TWIN instrument (Columbia, MD, United States). The instrument is equipped with a digital camera which allowed it to take photographic film. The instrument is also equipped with tilt stage and electron diffraction capabilities. The samples were prepared using a 10 μL aliquot sample of 1 mg of NOCNF in 10 mL DI water deposited on carbon coated Copper grids (300 mesh, Ted Pella Inc., Redding, CA, United States). The prepared grid was then stained with 2 wt. % aqueous uranyl acetate solution.

##### Atomic Force Microscopy (AFM)

AFM of NOCNF was performed using a Bruker Dimension ICON scanning probe microscope (Bruker Corporation, Billerica, MA, USA) equipped with a Bruker OTESPA tip (tip radius (max.) = 10 nm). In this measurement, a 10 µL of 0.005 wt. % NOCNF suspension was deposited on the surface of a silica plate, where the air-dried sample was measured in the tapping mode.

##### Zeta Potential Measurements

Zeta potential of the NOCNF sample was measured by Zeta probe Analyzer (Colloid Dynamics). This instrument consisted of a built-in titration setup equipped with pH electrode and Electrokinetic Sonic Amplitude (ESA) sensor probe. Before analyzing the sample, the pH electrode was calibrated using three different pH buffer standards (pH = 4.01, 7.01 and 10.01), followed by a standard titration solution. The ESA sensor was calibrated using the standard zeta probe polar solution (KSiW solution). Upon the completion of calibration test, the NOCNF suspension (0.26 wt. %, 250 mL) was filled in the sample holder, where the ESA sensor was then introduced into the sample under magnetic stirring to analyze the zeta potential. 

##### Dynamic Light Scattering (DLS)

The DLS of NRL sample was measured using Nano Brook 90 Plus particle size analyzer (Brookhaven, Holtsville, NY, USA) The DLS graph was set to lognormal plot and the data is an average of four total runs to obtain the polydispersity index (PDI).

##### Contact Angle Measurement

Static contact angle of NRL and composite films prepared by NOCNF and NRL were measured using the FDS-contact angle measurement instrument (Model no. OCA 15 EC). Films of NRL alone and composite films containing NRL and NOCNF were prepared by solvent casting method. A flat portion of film was cut and fixed onto sample holder. A 10 µL drop of deionized water was dropped onto film through a syringe needle operated automatically by syringe pump. The contact angle was measured 20 s after the drop casting to ensure that the water droplet reached its equilibrium position.

##### Tensile Test

The tensile data was obtained using the INSTRON model 4442 device (Instron, Norwood, MA, USA). Clamps were calibrated with a 1.75 cm gap and rectangular samples of 2 cm wide by 5 cm long were clamped evenly in the device. Elongation rate was set to 60 mm/min with data recording every second. Each data plot obtained is an average of three sets of experiments.

##### Scanning Electron Microscopy (SEM)

A Zeiss LEO 1550 SFEG-SEM instrument (White Plains NY, USA) was used to record SEM images of the samples. The instrument was comprised of an in-lens secondary electron detector in addition to the standard E-T detector, and a Rutherford backscatter electron detector. It was also equipped with an EDS (energy dispersive X-ray spectroscopy) system, provides elemental compositions and X-ray maps of the various phases of the materials examined. Images of surface morphology of NRL and NRL composite films were taken to observe the NRL film surface change on addition of NOCNF.

## 3. Results and Discussion

### 3.1. Characterization of NOCNF

**Figure 1:**
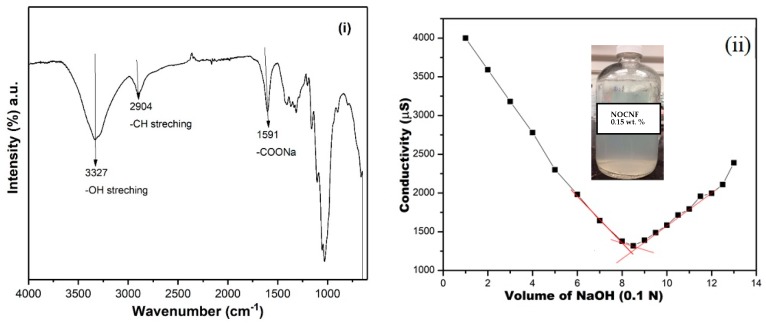
(**i**) Fourier transform infrared spectrometry (FTIR) of carboxycellulsoe nanofibers (NOCNF) and jute fibers, and (**ii**) conductometric titration graph to determine the carboxylate group on NOCNF (volume of NaOH consumed = 0.705 mL), inset the photograph of NOCNF suspension.

The characterization on the surface functionalization of NOCNF extracted from untreated jute fibers was first carried out by FTIR and conductometric titration. Figure 1i demonstrates the FTIR spectra of jute fibers and prepared NOCNF. The characteristic peaks of cellulose are ascribed at (i) 3327 cm^−1^ to O–H stretching vibrations, and at (ii) 2904 cm^−1^ to CH and CH_2_ stretching. The prominent peak in NOCNF at 1591 cm^−1^ presented the carboxylate groups (–COONa) appeared in NOCNF, which represents the oxidation of anhydroglucose unit at C6 position. Additional peaks at 1372, 1150, 1100, and 1030 cm^−1^ were due to stretching and bending vibrations in glycosidic bonds in cellulose. Other peaks in the FTIR of jute fibers such as 1512; 1732, 1456, 1235 and 808 cm^−1^ are because of aromatic symmetrical streching of C=C bonds in the lignin and in hemicellulose units respectively. Interestingly, the peaks belonging to lignin and hemicellulose completely disappear or significantly reduce in NOCNF, indicating that the nitro-oxidation was resonably effective in removing the lignin and hemicellulsoe impurities from raw jute fibers. The quantitative determination of lignin and hemicelulose in NOCNF was also perfomed to find the exact amount of lignin and hemicellulsoe, which is explained in the next section.

The quantitative determination of carboxylate groups in NOCNF was performed by conductometric titration method. The following equation (Equation (1)) was used to determine the degree of oxidation (*DO*):

(1) $$DO=\frac{MX\;\left(V_{2}-V_{1}\right)}{w}$$

where *M* is the molarity of NaOH in mol/L, *V_2_* and *V_1_* is the final and initial volume of NaOH in mL, *w* is the weight of the NOCNF dried fibers added in grams. 

The conductometric titration plot of NOCNF shown in Figure 1ii indicates its calculated DO value which is 0.94 mmol/g. This DO value shows that NOCNF contains a moderate degree of oxidation, which is further confirmed by zeta potential measurement. The zeta potential measurement demonstrates the presence of −115 ± 4 mV charge on the NOCNF surface. NOCNF showed good dispersion in water (inset photograph in Figure 1ii) because of the repulsion caused in between the fibers due to similar charges.

The lignin and hemicellulose analysis of raw jute fibers is presented in Table 1. It shows that the total hemicellulose (sugar content) and total lignin content (klason lignin (KL) + acid soluble lignin (ASL)) in the raw jute fibers was 68.9% and 17.55%, respectively. However, the total hemicellulose and lignin content in NOCNF was found as 65% and 1.94% respectively. The results indicate that the reasonable hemicellulose and residual lignin are still present in the NOCNF after the nitro-oxidation.

**Figure 2:**
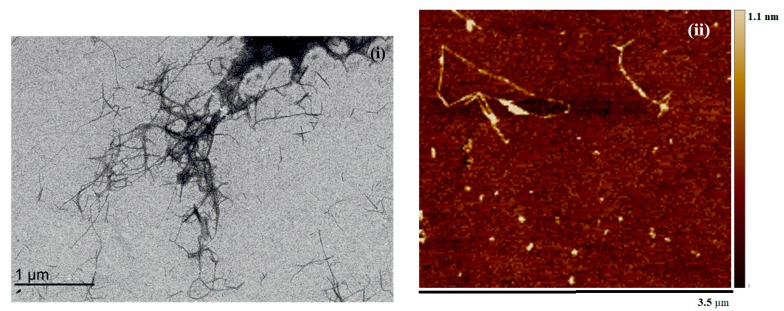
(**i**) Transmission electron microscopy (TEM) and (**ii**) atomic force microscopy (AFM) of NOCNF extracted from raw jute fibers.

The TEM image of NOCNF is shown in Figure 2i. The average fiber length observed for NOCNF was 524 ± 203 nm and width were in the range of 7 ± 2 nm. However, the AFM of the NOCNF indicated the average fibers thickness was 2.9 nm. In this study, the NOCNF obtained has greater width and thickness as compared to the cross section of most of the cellulose fibers where width is in the range of 4–5 nm and thickness ~1.5 nm [37]. This is probably due to the chosen nitro-oxidation conditions are relatively mild, where the presence of residual hemicellulose and lignin contents were still high.

**Figure 3:**
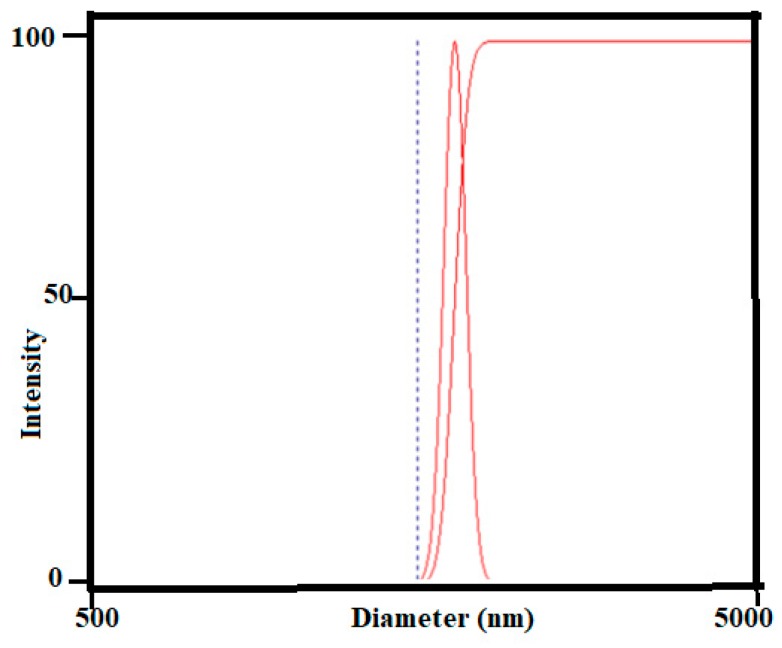
Dynamic light scattering (DLS) data of natural rubber latex (NRL) (average diameter = 637 nm with size polydispersity (PDI) = 0.005.

The dynamic light scattering (DLS) data measurement of NRL is presented in Figure 3. The data shows that the effective diameter of NRL molecule is 637 nm with polydispersity value of 0.005. This indicates that the NRL is composed of polyisoprene molecules with almost the same size. The TEM measurement of NOCNF indicated its fibers length in the range of 524 ± 203 nm, which is almost like the NRL particles’ size. We have assumed that similar sizes of two interacted molecules NOCNF and NRL will provide the better chances of their physical interaction. 

### 3.2. Characterization of Natural Rubber Latex (NRL) and Composite Films

**Figure 4:**
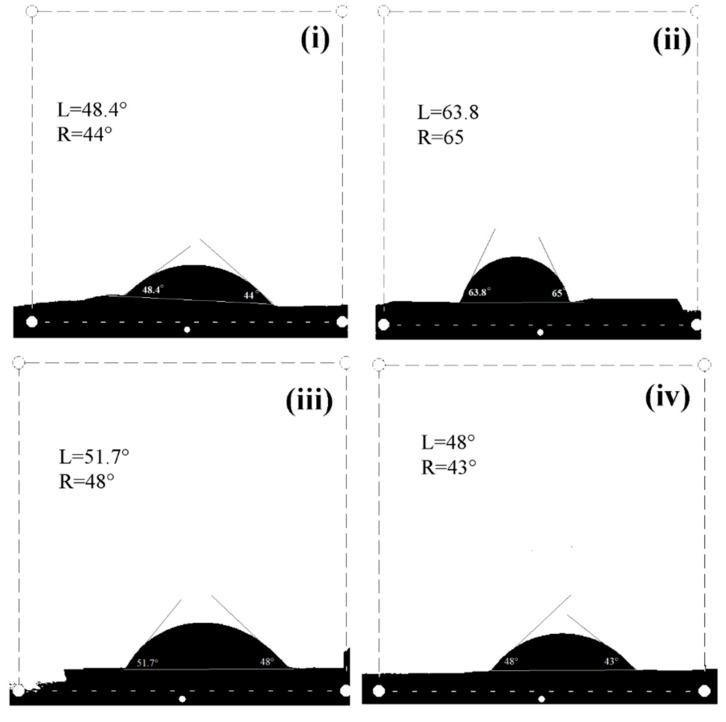
Contact angle measurements of (**i**) NRL film (control), and composite films made of NRL and NOCNF with varying concentration of NOCNF (**ii**) 0.1 wt. %, (**iii**) 0.2 wt. %, (**iv**) 0.4 wt.%.

The contact angle measurement was performed on the films prepared by NRL and the composite films made of NRL and varying content of NOCNF (0.1, 0.2 and 0.4 wt. %), which is shown in Figure 4. The contact angle measurement of pure NRL has shown the average angles (left = 63.8° and right = 65°) clearly indicate that it has hydrophobic surface. An earlier study of the chemically crosslinked NRL film (using potassium persulphate (KPS) as an initiator) reported that the contact angle should be in the range of 94°, which was clearly more hydrophobic than the noncrosslinked film in the present study [38]. However, the small addition of NOCNF (0.1 wt. %) into NRL has changed the average contact angle of composite membrane to be around 49.5 ° (left = 51° and right = 48°). The decrease in contact angle indicates the appearance of hydrophilic behavior in composite film because of the presence of NOCNF which has more hydrophilic groups such as hydroxyl and carboxylate. The further addition of NOCNF (0.2 and 0.4 wt. %) into NRL has further reduced the contact angle of composite films (e.g., film with 0.2 wt. % NOCNF: left = 48.4° and right = 44°; film with 0.4 wt. % NOCNF: left = 48° and right = 43°). Interestingly, not much change in the contact angle of composite films containing 0.2 and 0.4 wt. % of NOCNF was observed. This is probably because 0.2 wt.% is the overlapping concentration of NOCNF [31], where some aggregations might have occurred in the NRL matrix at and above overlapping concentration (i.e., 0.2 and 0.4 wt. %) of NOCNF, which resulted in decrease in hydrophobicity of the composite films.

### 3.3. SEM Images

**Figure 5:**
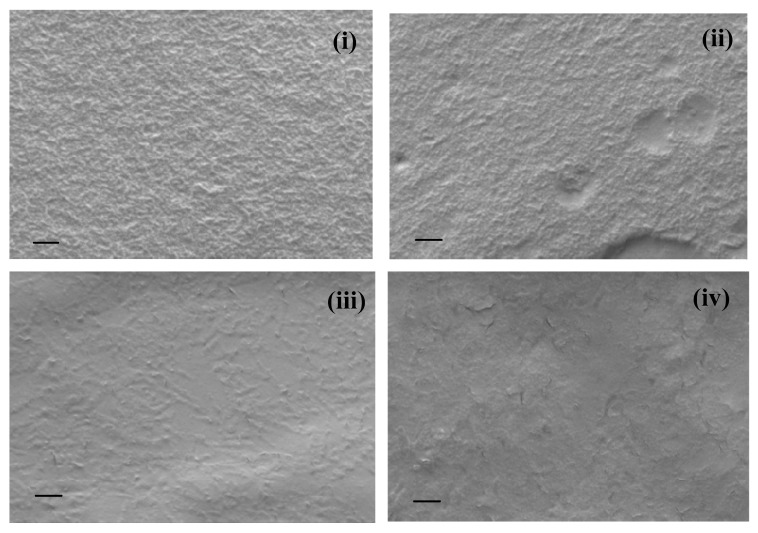
Scanning electron microscopy (SEM) images taken at scale bar = 200 nm on (**i**) NRL film (control), and composite films made of NRL and NOCNF with varying concentration of NOCNF (**ii**) 0.1 wt.%, (**iii**) 0.2 wt.%, (**iv**) 0.4 wt.%. Red circles indicate the cracks in the film.

SEM images of film made of NRL and composite films consisting of NRL and varying concentrations NOCNF are presented in Figure 5. The image of NRL in Figure 5i indicates that the film possesses uniform surface roughness. This was due to the agglomeration of similar granular shape NRL particles. This was consistent with the size characterization of NRL using the DLS technique (Figure 3, NRL possessed an average particle size of 640 nm with the polydispersity of 0.005). The morphology of NRL film on addition of 0.1 wt. % of NOCNF (nanofibers) has not shown any significant changes on the surface appearance. However, the addition of 0.2 wt. % and 0.4 wt. % nanofibers into NRL (Figure 5iii,iv) resulted in significant changes in surface appearance in terms of roughness.

The roughness of the latex film decreases on increasing the amount of nanofibers concentration above 0.1 wt. % that could be because of the well distribution of NOCNF with the latex particles. These data correlate well with the contact angle measurement, as the drastic decrease in contact angle from 63° to 43° was observed for the NRL film on addition of 0.1 and 0.2 wt. % of NOCNF. However, the cracks start beginning in composite films containing the (highest) 0.4 wt. % of NOCNF. The probable reason for the crack could be the phase separation in between NRL and NOCNF because of their corresponding hydrophobic and hydrophilic behavior. 

### 3.4. Mechanical Properties of Latex and Composite Films

**Figure 6:**
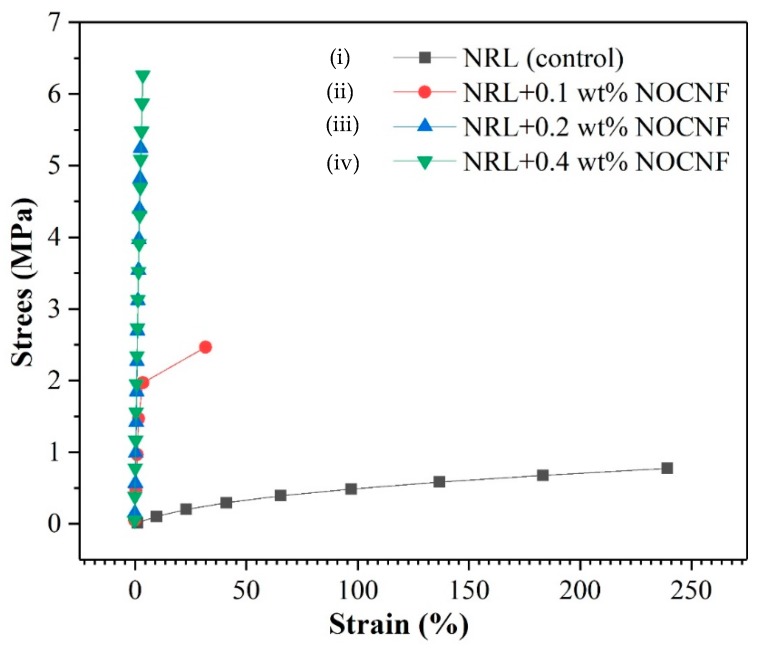
Stress–strain curves on (**i**) NRL film (control), and composite films made of NRL and NOCNF with varying concentration of NOCNF (**ii**) 0.1 wt.%, (**iii**) 0.2 wt.%, (**iv**) 0.4 wt.%.

The mechanical properties of NRL and composite films containing NRL and varying concentrations of NOCNF is shown in Table 2. The stress–strain curve was plotted for all the composite films and is presented in Figure 6. The stress on the y-axis was calculated by dividing the load by the cross-sectional area of the film. The thickness and length of the film is measured using a caliper and all nanocomposites showed the same thickness of 0.08 cm^2^. There could be a slight variation of thickness due to the increased amount of NOCNF added, but the difference would be negligible since the NRL solid amount contributes to most of the sample’s volume. The stress–strain curves for the NRL and composite films also indicates their ultimate tensile strength (UTS).

It was found that the NRL film exhibited the UTS value of 0.77 Mpa. However, the addition of NOCNF increased the UTS value of the film quite notably (e.g., the films with 0.1, 0.2 and 0.4 wt. % of NOCNF showed the UTS value of 2.5, 5.2 and 6.2 MPa, respectively). We note that the ultimate tensile strength reported for the pure NOCNF extracted from jute fibers using the nitro-oxidation method was 108 MPa [31] and the chemically crosslinked NRL film typically exhibited the UTS value of 27 Mpa [39]. In the above study [39], vulcanized (chemically crosslinked) NRL was reinforced by addition of cellulose nanocrystals (CNC) where the 3 wt. % of CNC was found to be most effective to increase the ultimate tensile strength of NRL (by about 29%). In this study, the authors have used the term cellulose nanofibers to describe nanocellulose isolated from coconut spathe using the acid hydrolysis method. We believe that the length of such nanocellulose particles extracted by the acid hydrolysis approach should be shorter, the cross-sectional dimensions larger and the crystallinity higher than those in NOCNF, and they should be termed CNC. It is interesting to note that the overlap concentration of CNC usually varies between 1.5 wt. % to 3 wt. %, depending on the source of the biomass [40]. We hypothesize the ultimate tensile strength of vulcanized CNC-NRL also takes place near the overlap concentration of CNC. 

On the other side, the maximum elongation (*λ_max_*, %) observed in the tested films showed an opposite trend. For example, the pure NRL film exhibited *λ_max_* at about 234 %, where 0.1 wt.% of NOCNF in the composite film decreased the *λ_max_* value to 31.4 %. The increase in the NOCNF content further decreased the *λ_max_* value (e.g., 0.2 and 0.4 wt. % of NOCNF films showed the *λ_max_* value of around 2.5% and 3.5%), rendering the films to be quite brittle. These results were consistent with the SEM images (Figure 5), which showed that the addition of NOCNF decreased the roughness of the NRL film and increased the content of cracks owing to phase separation between NOCNF and NRL.

**Figure 7:**
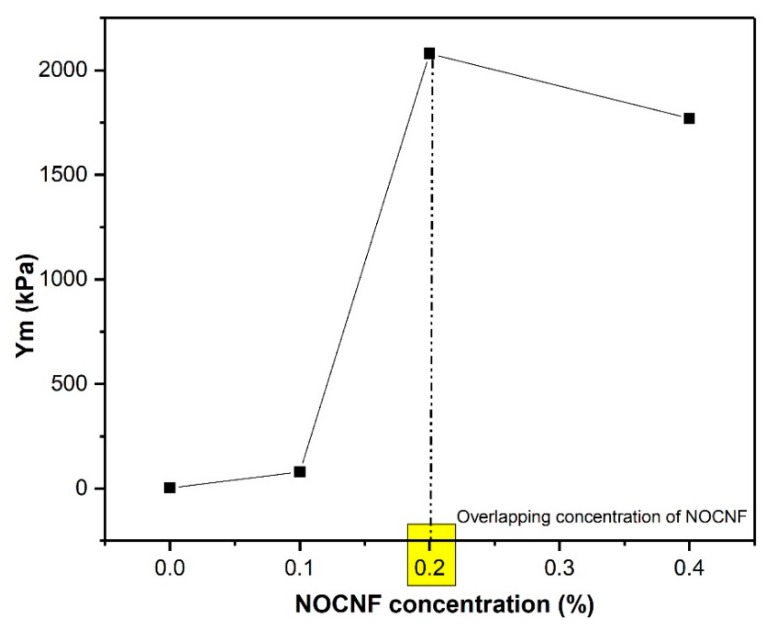
Graph represents the relationship between the Young’s modulus (kPa) and the NOCNF concentration in the composite films.

The ductile–brittle transition was also noticeable from the Young’s modulus (Ym) evaluation shown in Figure 7. The pure noncrosslinked NRL was very ductile, showing an Ym value of merely 3.5 KPa, where 0.1 wt. % of NOCNF addition increased the Ym value to 79.6 KPa and 0.2 wt. % of NOCNF addition increased the Ym value maximum to 2080 kPa. The further increase of NOCNF content decreased the Ym value to 1770 kPa. As a result, the higher NOCNF content would not lead to any property enhancement, where the best content of NOCNF addition appeared to occur near its overlap concentration (0.2 wt. %).

## 4. Conclusions

This study showed that the nitro-oxidized carboxycellulose nanofibers (NOCNF) extracted from raw jute fibers could be incorporated into the NRL matrix to increase the mechanical properties even in a nonvulcanized state. The optimal amount of the NOCNF for the overall property improvement seems to take place around the overlap concentration of NOCNF (around 0.2 wt. %). This is not surprising as the overlap concentration of nanocellulose represents the transition point from a viscous state to a gel state. Addition of NOCNF into non-vulcanized rubber has changed the NRL film from elastic to brittle. The more schematic study on latex composite preparation can explore the use of these inexpensive and sustainable nanofibers material into the preparation of other rubber-based (e.g., Guayule) composite materials.

## Figures and Tables

**Table 1:** Characteristic of carboxycellulsoe nanofibers (NOCNF) obtained from jute.

Sample	Carboxylate Content (mmol/g)	Zeta Potential (mV)	Residual Lignin (%) KL/ASL ^a^	Residual hemicellulose (%)	Length/Width (nm)	Thickness (nm)
NOCNF	0.94	−115 ± 4	0.58/1.36	65	524 ± 203/ 7 ± 2	2.9

^a^ KL = klason lignin, ASL = acid soluble lignin.

**Table 2:** Young’s modulus (Ym), ultimate tensile strength (UTS), and maximum elongation (*λ_max_*). Each value is the average of three replicates samples. NRL—pure natural rubber latex, NRL 0.1—NRL containing 0.1 wt. % of NOCNF; NRL 0.2—NRL containing 0.2 wt. % of NOCNF; NRL 0.4—NRL containing 0.4 wt. % of NOCNF.

Sample	Ym (kPa)	UTS (MPa)	*λ_max_* (%)
NRL	3.3	0.77	234
NRL 0.1	79.6	2.5	31.4
NRL 0.2	2080	5.2	2.5
NRL 0.4	1770	6.2	3.5

## Supplementary Materials

The following are available online at https://www.mdpi.com/2079-4991/10/4/706/s1.
