# Supplementary material for: Reinforcement of Natural Rubber Latex Using Jute Carboxycellulose Nanofibers Extracted Using Nitro-Oxidation Method
Source: Nanomaterials (Basel). 2020 Apr 8;10(4):706. doi: 10.3390/nano10040706 (PMC7221719; doi:10.3390/nano10040706)
Supplement: Supplementary file 1 [file nanomaterials-10-00706-s001.pdf]

nanomaterials

Supplementary Materials

Reinforcement of Natural Rubber Latex Using Jute Carboxycellulose Nanofibers Extracted using Nitro-oxidation Method

Sunil K. Sharma,[1] Priyanka R. Sharma,[1*] Simon Lin,[1] Hui Chen,[1] Ken Johnson,[1] Ruifu Wang,[1] William Borges,[2] Chengbo Zhan,[1] Benjamin S. Hsiao[1*]

[1] Department of Chemistry, Stony Brook University, Stony Brook, NY 11794-3400
[2] Roslyn High School, Roslyn, NY-11576

* Correspondence: priyanka.r.sharma@stonybrook.edu Tel: +1631-5423506; benjamin.hsiao@stonybrook.edu; Tel: +1-631-6327793

Analytical Procedure for Lignocellulsoe and Sugars include:

The procedure for analysis of the jute fiber and NOCNF is shared by Celignis Analytical. It includes following procedures.

1. Acid Hydrolysis of the Sample

The acid hydrolysis takes place on the biomass sample with no prior extraction carried out at Celignis. If previous steps involving the extraction of the sample have taken place, then the extracted material is used for the acid hydrolysis. In the case where three different types of extracted material exist (water-extracted, ethanol-extracted, and water- then ethanol-extracted) then the sample that has undergone both water and ethanol extraction is typically used for acid hydrolysis.
Acid hydrolysis of the sample involves following steps:

1. The moisture content of the sample is determined, in duplicate.
2. Approximately 300 mg (with the exact weight noted) of the sample is added to a pressure tube.
3. 3.00 mL of 72% H_2SO_4 is added by means of an automatic titrator, the weight of the acid added is noted.
4. The sample is mixed thoroughly with the acid using a glass rod, care is taken that no sample stays adherent to the sides of the tube, but instead stays in contact with the acid.
5. The tube is transferred to a water bath that is maintained at 30 °C.
6. Steps 2-5 are repeated for the duplicate sample.
7. Every 10 minutes the glass rod for each pressure tube is stirred so that the acid reaches all parts of the sample and complete hydrolysis occurs. This is a crucial step.
8. Exactly one hour after it is placed in the water bath the pressure tube is removed and placed on a scales and 84 mL of water added (with the weight of the added water recorded). Any acid/sample on the rod is removed from the rod at this point using this water.
9. A lid is screwed on the tube and the tube is inverted several times to ensure thorough mixing of the acid.
10. Two sugar recovery solution (SRS) pressure tubes are prepared in order to monitor the sugar-loss associated with the second stage-hydrolysis. This involves the following steps:
(a) 348 μL of 72% H_2SO_4 is added to a test tube containing a solution containing a known weight (approximately 10 g) of a sugar standard. This standard should be of a similar sugar composition to that expected of the samples being analyzed. The acid and sugar solution are thoroughly mixed.
(b) The sugar-acid mixture is transferred to a pressure tube which is then sealed.
11. All SRS and sample pressure tubes are placed in an autoclave which is run at 121 °C for 60 minutes.
12. After the temperature in the autoclave drops to under 80 °C the tubes are removed and are left (closed) in the lab until they reach room temperature.

13. The hydrolysates are then filtered (using vacuum suction) through filter crucibles of known weight and the resulting filtrate is stored.
14. Any residual solids are washed out from the tube using deionized water until all the acid insoluble residue resides on the filter crucible.

2. Determination of Acid Soluble Lignin (ASL)

The following steps are involved in the determination of the Acid Soluble Lignin content:
1. The hydrolysate (filtrate) from the Acid Hydrolysis Stage, is quantitatively transferred using a pipette to a 1 cm path-length (3 mL volume) quartz cuvette.
2. The UV-Visible (190-520 nm) transmission spectrum of the sample is then obtained using the HP 8452A diode-array spectrophotometer.
3. If the absorbance at a given wavelength is outside of the linear region (considered to be between 0.7 and 1.0 absorbance units) then the sample is diluted with water until the value falls within this region.
3. This dilution and UV-Visible analysis is carried out twice for each hydrolysate, meaning that each sample will have four spectra collected (two spectra for each of the duplicate hydrolysates).
4. The Acid Soluble Lignin content is then calculated based on the absorbance value at 205 nm, the dilution factor, and a given absorptivity constant.

3. Hydrolyzate dilution and Storage

Following the acid hydrolysis step, the hydrolysate (the filtrate from the vacuum filtration) is diluted by a factor of 5 using a fucose-in-water solution. Fucose is the internal standard that is used in the chromatographic analysis of the hydrolysate. Following this dilution the samples are either immediately put on the chromatography system or stored in a freezer for future analysis.

4. Gravimetric Determination of Klason Lignin

The following steps are involved in the gravimetric determination of the Klason lignin, Acid Insoluble lignin and Acid Insoluble Ash contents:
1. The filter crucibles from the Acid Hydrolysis Step are placed in an oven overnight and dried at 105 °C.
2. These crucibles are then weighed to determine the acid insoluble residue content.
3. The crucibles are then placed in a Nabertherm L-240H1SN muffle furnace and the following program used:
(i) Ramp from room temperature to 105 °C.
(ii) Hold at 105 °C for 12 minutes.
(iii) Ramp to 250 °C at 10 °C /minute.
(iv) Hold at 250 °C for 30 minutes.
(v) Ramp to 575 °C at 20 °C / minute.
(vi) Hold at 575 °C for 180 minutes.
(vii) Allow temperature to drop to 105 °C.
(viii) Hold at 105 °C until the samples are removed.
4. The fiter crucibles are then weighed and the acid insoluble residue, and acid insoluble ash content calculated.
5. The klason lignin content is then calculated by subtracting the acid insoluble ash content from the acid insoluble residue content.

5. Chromatographic analysis of the Hydrolysate
